# Precision bacterial immunotherapy: an integrated mechanistic taxonomy and translational roadmap against antimicrobial resistance

**DOI:** 10.3389/fimmu.2025.1675682

**Published:** 2025-12-19

**Authors:** Hamdi Al-Azzani, Djandan Tadum Arthur Vithran, Hanane Aliouat, Wenhu Zhou, Xiaoqiang Mao

**Affiliations:** 1 Xiangya School of Pharmaceutical Sciences, Central South University, Changsha, Hunan, China; 2 Department of Orthopedics, Xiangya Hospital, Central South University, Changsha, Hunan, China; 3 National Clinical Research Center for Geriatric Disorders, Xiangya Hospital, Central South University, Changsha, Hunan, China; 4 Hunan Key Laboratory of the Research and Development of Novel Pharmaceutical Preparations, School of Pharmaceutical Science, Changsha Medical University, Changsha, China; 5 The Quzhou Affiliated Hospital of Wenzhou Medical University, Quzhou People’ s Hospital, Quzhou, Zhejiang, China

**Keywords:** bacterial immunotherapy, antimicrobial resistance, monoclonal antibodies, CRISPR therapeutics, nanoparticle delivery, host–pathogen interaction

## Abstract

An integrated, host-directed therapeutic strategy is urgently required to outpace the accelerating threat of antimicrobial resistance (AMR), because pathogen-centred antibiotics are losing efficacy worldwide. The growing threat of antimicrobial resistance has made traditional antibiotics less and less effective, and it has also shown that our pathogen-centered treatment model has systemic flaws. Bacterial immunotherapy offers an alternative that is directed at the host. Still, its many forms, such as innate immune agonists, monoclonal antibodies, engineered cells, CRISPR-based antimicrobials, and aptamer-guided nanoplatforms, have mostly been looked at separately. We put these different approaches together in this narrative review to create a new mechanistic taxonomy that shows how they can be used together to break down biofilms, stop efflux pumps, and get rid of intracellular reservoirs. We then put each modality on a translational continuum, from bench-top proof-of-concept to late-stage trials, and find the most essential delivery, safety, and regulatory problems. Lastly, we describe a precision-first vision that uses multi-omics profiling and theranostic platforms to help with patient stratification, adaptive dosing, and real-time monitoring. This review shows a straightforward way to turn narrative insights into context-sensitive, long-lasting interventions that will work with and maybe even change the future of infectious disease medicine by co-developing immunotherapeutic strategies with advanced diagnostics and stewardship frameworks.

## Graphical Abstract

**Figure:**
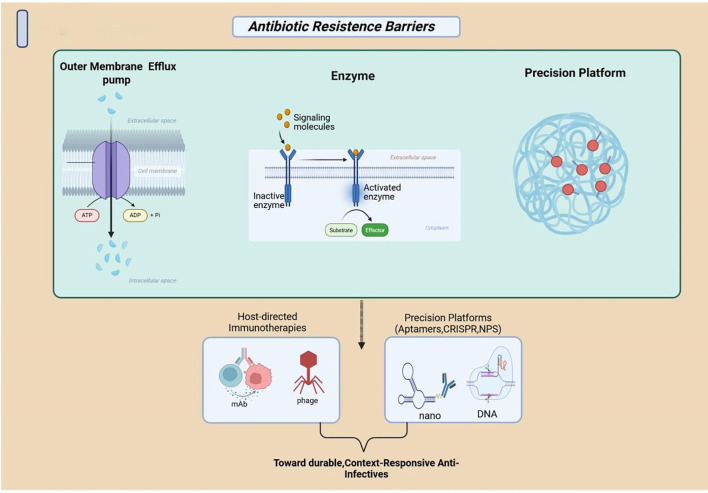
This diagram shows the many problems that make traditional antibiotics less effective. These include ATP-driven outer-membrane efflux pumps that actively remove antimicrobial agents, inducible enzymatic pathways that deactivate drugs when they receive signals from outside the cell, and the fact that passive delivery vehicles don’t work well enough. It also shows the convergent immunotherapeutic and precision-platform solutions that have been made to solve these problems. The lower panel shows how host-directed modalities (monoclonal antibodies and bacteriophages) use both innate and adaptive immune mechanisms to kill pathogens and break up biofilms. It also shows how precision technologies (aptamer-guided nanoparticles and CRISPR-based systems) can target specific sequences and release them at the right time in the infection environment. These complementary strategies work together to create a translational roadmap for long-lasting, context-sensitive anti-infective interventions that can get around multidrug-resistance mechanisms.

## 1 Introduction

Bacterial infections remain a chronic and severe concern in contemporary medicine, imposing a substantial strain on global healthcare systems. These infections are induced by a varied assemblage of unicellular prokaryotes that lack nuclear membranes and exhibit considerable morphological and structural variation. Gram staining and biochemical profiling are crucial for the identification of bacterial species and the prediction of antibiotic susceptibility (1, 2).

Commensal bacteria, particularly those constituting the human microbiota in the gastrointestinal tract, dermis, and mucosal surfaces, play a crucial role in essential physiological functions including immunological modulation, nutrition metabolism, and pathogen protection (3). When microbial defenses are compromised or virulent strains penetrate host barriers, pathogenic bacteria can induce severe infections. These infections pose particular risks to neonates, the elderly, and individuals with comorbidities or immunosuppression, frequently resulting in considerable morbidity, mortality, and healthcare costs (4, 5).

A significant hazard intensifying the challenge of infectious diseases is the swift and worldwide increase of AMR. This phenomenon results from genetic adaptability and selective pressure, leading bacterial populations to gradually develop resistance to different medication classes. Multidrug-resistant (MDR), extensively drug-resistant (XDR), and pandrug-resistant (PDR) organisms are currently widespread in both healthcare and community environments (6). The improper use of antibiotics in clinical settings and agriculture has exacerbated this tendency, compromising the efficacy of current treatments (7). The mechanisms of resistance include horizontal gene transfer, efflux pumps, biofilm formation, enzymatic drug inactivation, and target alteration, hence rendering numerous standard antibiotics ineffective (8, 9).

Notwithstanding the gravity of this issue, the development pipeline for novel antibiotics has markedly decelerated. Scientific constraints, regulatory obstacles, exorbitant costs, and insufficient market incentives have all resulted in minimal novel therapeutic advancements over the past two decades (10). This stagnation has generated an urgent need for alternative, practical strategies to control bacterial infections without speeding up resistance.

Bacterial immunotherapy is a promising and increasingly well-known solution. It is a new approach that changes how we treat infections by changing the host’s immune system instead of directly targeting bacteria. Immunotherapy uses natural and adaptive immune mechanisms, like pathogen recognition, phagocytic clearance, and immunological memory, to control and get rid of bacterial pathogens. This is different from regular antibiotics (11). Immunotherapies may help stop the evolution of resistance by making people less dependent on direct bactericidal agents. This could make treatments last longer.

Recent progress in bacterial immunotherapy builds on discoveries made in the field of cancer treatment. Some promising strategies are monoclonal antibodies, immune checkpoint inhibitors, cytokine therapies, and vaccine-based treatments. Many of these are based on successful cancer immunotherapies and are now being looked into for infectious diseases (12) These immunotherapies can also be made even more precise, specific, and effective by combining them with cutting-edge technologies like aptamer-functionalized nanoparticles, CRISPR-based antimicrobials, and engineered immune cells like CAR macrophages. These new methods offer new ways to get around the problems with traditional antibiotics, such as getting through biofilms, killing pathogens inside cells, and reducing side effects.

Precision Bacterial Immunotherapy (PBI) denotes any single host-directed modality such as a toxin-neutralising antibody, a CRISPR phagemid, or an engineered phagocyte applied on its own. Integrated Precision Strategies (IPS) refer to deliberate combinations of two or more PBI modalities (for example, an antibody co-delivered with an aptamer-guided nanocarrier) that act synergistically to neutralise multiple bacterial defences in parallel.

**Figure 1:**
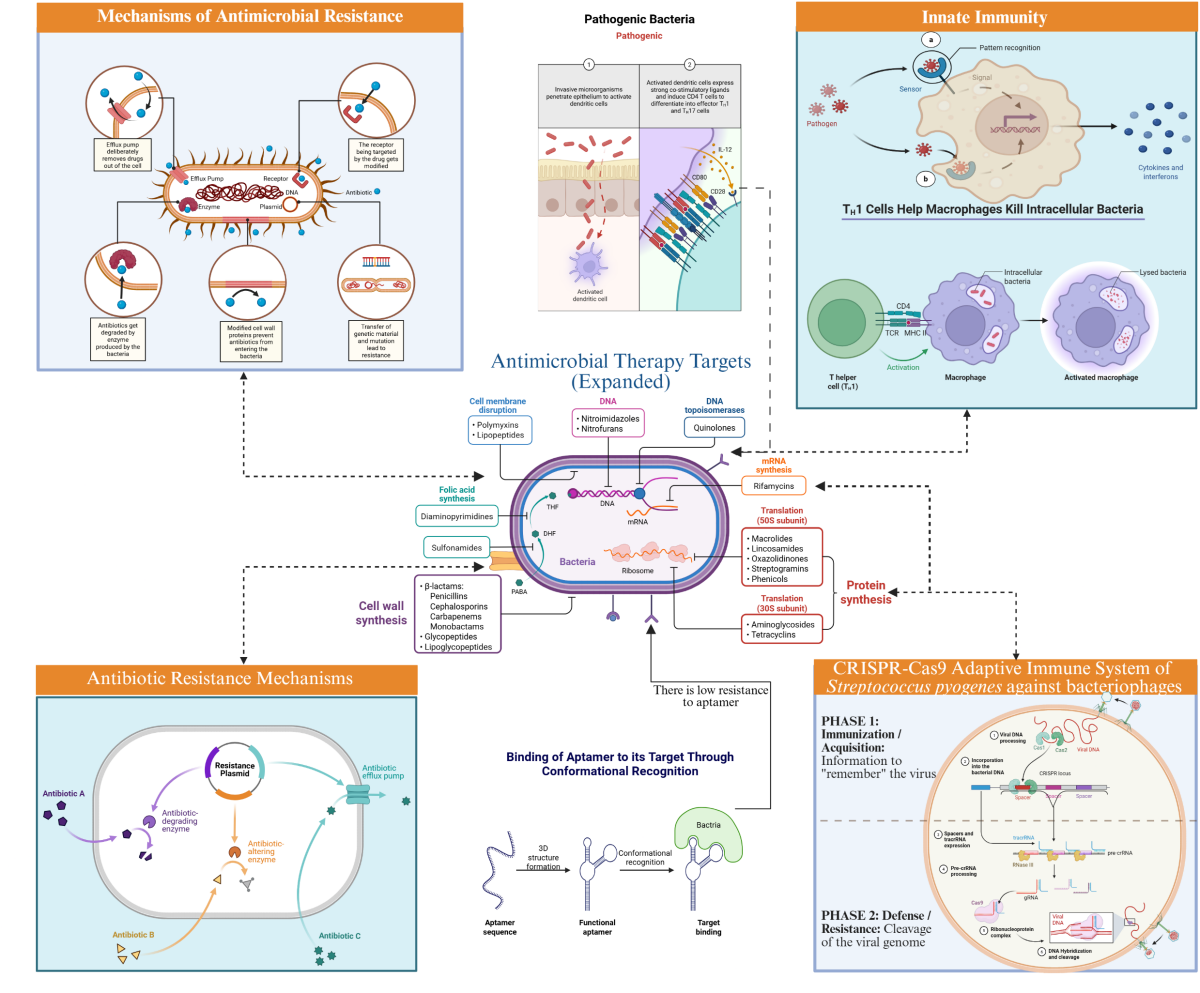
Mechanistic taxonomy of precision bacterial immunotherapy and integrated IPS. This schematic integrates the full spectrum of host-directed antibacterial modalities into a single, three-tier framework that maps (i) Precision Bacterial Immunotherapy (inner ring)—stand-alone toxin-neutralising monoclonal antibodies, innate-immune agonists, checkpoint inhibitors, cytokines, and vaccine platforms—to (ii) Integrated Precision Strategies (middle ring) in which complementary technologies (CRISPR/Cas phagemids deleting resistance cassettes, CAR-engineered phagocytes targeting biofilms, and aptamer-guided or lipid-nanoparticle carriers concentrating drugs at infection foci) are rationally co-deployed, and finally to (iii) Bacterial Defence Barriers Overcome (outer ring) including toxin production, biofilm formation, intracellular persistence, and horizontal gene-transfer–mediated resistance. Radial arrows indicate the primary mechanism by which each modality dismantles a specific bacterial defence, while concentric colour bands denote the translational stage—pre-clinical (light), early-phase clinical (medium), or late-phase/approved (dark). The infographic succinctly illustrates how layering complementary host-directed interventions can neutralise multiple bacterial survival strategies simultaneously, thereby offering a roadmap for designing next-generation IPS regimens that are both mechanistically synergistic and clinically actionable.

Here, we introduce a mechanistic taxonomy that organises single-agent PBI and multi-component IPS by the bacterial defences they target and the host pathways they engage (**Figure 1****).** This schema provides a visual map linking modalities to biofilms, efflux systems, intracellular persistence, and immune-evasion/toxin axes, and it anchors the modality-specific sections that follow.

This review’s goal is to look at the current state of bacterial immunotherapy systematically, including how it works with new technologies like nanomedicine and aptamer-based delivery systems. We want to talk about how these therapies work, how they have progressed in the clinic, and the challenges that come with moving them from the lab to the hospital. This review will not cover antiviral or antifungal immunotherapies; instead, it will only look at bacterial targets and host-pathogen interactions that are important for AMR. In the end, this paper aims to identify key gaps in our knowledge, outline clear pathways for translating that knowledge into practice, and propose new areas of research to accelerate the clinical application of bacterial immunotherapy.

## 2 Methods

### 2.1 Eligibility criteria

For this narrative review, studies were selected based on their relevance to the topic of bacterial immunotherapy targeting AMR. Eligible studies were those that explored bacterial immunotherapies, including monoclonal antibodies, CRISPR-based antimicrobials, engineered immune cells, and aptamer-guided nanoplatforms. We included studies from a wide range of sources, including preclinical research (e.g., *in vitro* studies, animal models) and clinical trials (from Phase I to Phase III). Only peer-reviewed publications published in English between 2000 and 2025 were considered. Studies on viral, fungal, or non-bacterial pathogens were excluded, as were those that did not directly contribute to our understanding of immunotherapeutic approaches in bacterial infections.

### 2.2 Information sources and search strategy

To identify relevant literature, we have handled the search process within three scientific databases, including PubMed, Scopus, and Web of Science. The main purpose of exploring the three databases cited above is to build a preliminary overview of the afforded literature and understand the principle points to review. The search queries were established to collect illustrative scientific works correlated to each section of the pre-established summary of this article. Assessment of articles’ relevance was performed by two different authors for each section; the writer and the reviewer, based on the selection criteria established previously. Full-text articles were retrieved and discussed for records that either not meeting all inclusion criteria or lacked sufficient information. Disagreements during either the screening or full-text evaluation were resolved by discussion and consensus. If consensus could not be reached, a third senior reviewer author was consulted for final adjudication. This multi-reviewer process was used to minimize selection bias and improve methodological rigor.

Depending on the related section, queries could vary. The query strategy was similar in core for each database, including same keywords with differences imposed by the particularities of each advanced search bar of each platform (MeSH words for PubMed, Boolean operators for PubMed and Scopus, refining strategies for Web of Science). An example of searching strategies for establishing the state of art about bacterial resistance and conventional therapies in PubMed search included as follows:

“Bacterial Infections”[MeSH] OR “Drug Resistance, Bacterial”[MeSH] OR “Drug Resistance, Microbial”[MeSH].

For the cutting-edge therapies review the query was:

“Antibacterial Agents”[MeSH] AND (“Immunotherapy”[MeSH] OR “Monoclonal Antibodies”[MeSH] OR “CRISPR-Cas Systems”[MeSH] OR “Nanoparticles”[MeSH] OR “Aptamers”[MeSH] OR “Host-Pathogen Interactions”[MeSH] OR “Biofilms”[MeSH] OR “intracellular” OR “efflux pump” OR “toxin neutralization” OR “engineered immune cells”) AND (“2000/01/01”[PDAT]: “2025/12/31”[PDAT]).

As demonstrated below, the search strategy was refined iteratively to identify the most relevant studies for each section topic. These detailed search strings are provided in **Table 1** to ensure that the strategy is fully reproducible.

**Table 1:** Key MeSH terms for search strategy.

Concept area	MeSH terms used	Rational
Bacterial infections	“Bacterial Infections”[MeSH]	Core disease category
Antimicrobial resistance	“Drug Resistance, Bacterial”[MeSH]	Captures AMR mechanisms
Immunotherapy	“Immunotherapy”[MeSH]	Core therapeutic category
Monoclonal antibodies	“Antibodies, Monoclonal”[MeSH]	Specific host-directed therapy
CRISPR antimicrobials	“CRISPR-Cas Systems”[MeSH]	Gene-editing antimicrobials
Nanomedicine	“Nanoparticles”[MeSH], “Nanomedicine”[MeSH]	Delivery platforms
Aptamers	“Aptamers”[MeSH]	Targeted binding technologies
Host–pathogen interaction	“Host-Pathogen Interactions”[MeSH]	Mechanistic scope

This review is based on a narrative methodology, which exposes the collected knowledge to several bias and limitations. Although a structured and reproducible search strategy was employed, the methodological standards of systematic reviews were not strictly applied. As a result, possible selection bias cannot be fully excluded.

The literature reviewed herein explores diverse immunotherapeutic platforms used against bacteria, including monoclonal antibodies, CRISPR-based antimicrobials, engineered immune cells, and aptamer-guided nano platforms. The variation of methodological quality and clinical maturity brings heterogeneity limits and hinders direct comparisons across studies.

Furthermore, many of the emerging technologies discussed in this review remain in early preclinical stages, with limited sample sizes, which restricts broadening of the findings. The rapidly evolving nature of antimicrobial resistance also means that some clinical findings may become outdated quickly.

Finally, despite these constraints, the narrative review approach allows an integrative and mechanistic synthesis of the topic and offers an overview of the state of art about the developments in antibacterial treatments.

### 2.3 Selection of sources of evidence

The articles identified in the search process were initially screened based on their title and abstract. Only those that clearly addressed bacterial immunotherapy modalities (such as monoclonal antibodies, CRISPR, and aptamer-based systems) and their role in AMR were selected for full-text review. Studies that did not focus on immunotherapy approaches to treat bacterial infections or that lacked relevant data on efficacy, safety, and clinical translation were excluded. Two independent reviewers performed the selection process to minimize bias, with disagreements resolved through consensus.

### 2.4 Data extraction and charting process

Data were extracted from each included study using a custom data extraction form, which included key information such as author, year, study design, immunotherapy modality, mechanism of action, and clinical outcomes (e.g., efficacy in bacterial clearance, reduction in resistance mechanisms, etc.). We did not perform a full critical appraisal of the studies, as this is typically not required for a narrative review. Instead, we focused on synthesizing the key findings of each study and discussed the methodological quality where relevant. If the methodology or sample sizes were unclear or limited, this was noted in the synthesis of results.

### 2.5 Synthesis of results

We synthesized the results narratively, categorizing the findings by immunotherapy modality (e.g., monoclonal antibodies, CRISPR-based systems, etc.). The synthesis aimed to identify trends in efficacy, safety, delivery challenges, and regulatory concerns associated with each modality. We also highlighted knowledge gaps in the current research, such as the limited clinical trial data for certain therapies and the need for more robust *in vivo* studies. The synthesis was aimed at providing a comprehensive overview of the state of bacterial immunotherapy in AMR, offering insights into the potential clinical applications and the future directions for research in this field.

## 3 Classification of antibiotics and their mechanisms

Antibiotics work by targeting specific processes in bacteria, such as cell wall biosynthesis, protein translation, nucleic acid metabolism, and membrane integrity. However, because resistance builds up so quickly, these mechanisms need to be constantly reevaluated to help find new targets and improve drugs (6, 8). β-Lactams (penicillins, cephalosporins, carbapenems) bind penicillin-binding proteins to block peptidoglycan cross-linking, while glycopeptides (vancomycin) sequester D-Ala-D-Ala termini to obstruct transglycosylation; both remain cornerstones against Gram-positives despite pervasive β-lactamases and PBP2a alterations (13, 14). Polymyxins (colistin) and lipopeptides (daptomycin) are membrane-active agents that break down the envelopes of Gram-negative and Gram-positive bacteria, respectively. They are effective as a last resort, but they can be toxic to the kidneys and resistant to mcr-mediated resistance (15, 16). See **Table 2** for a summary of classes, mechanisms, and resistance pathways.

**Table 2:** Overview of antibiotic classes: mechanisms, resistance mechanisms, and therapeutic implications.

Antibiotic class	Examples	Mechanism of action	Spectrum	Resistance mechanisms	Key clinical notes	References
β-Lactams	Penicillin G, Amoxicillin, Cefazolin, Meropenem	Inhibit cell wall synthesis via PBPs, blocking peptidoglycan cross-linking	Broad (Gram+/Gram–)	β-lactamases, altered PBPs, porin loss	Carbapenems active vs ESBLs; resistance rising	(8, 14)
Glycopeptides	Vancomycin, Teicoplanin	Bind D-Ala-D-Ala, inhibit peptidoglycan transglycosylation	Gram-positive (MRSA, VRE)	Altered binding site (VanA/VanB genes)	Reserved for resistant Gram+; nephrotoxicity risk	(14)
Aminoglycosides	Gentamicin, Amikacin	Bind 30S ribosomal subunit, cause mRNA misreading	Aerobic Gram-negative	Enzymatic modification, efflux, ribosome methylation	Nephrotoxic, ototoxic; post-antibiotic effect	(17)
Tetracyclines	Doxycycline, Minocycline	Bind 30S, block aminoacyl-tRNA attachment	Broad (Gram+/Gram–, atypicals)	Efflux pumps, ribosome protection proteins	Avoid in pregnancy; dental discoloration	(18–20)
Macrolides	Erythromycin, Azithromycin	Bind 50S, inhibit translocation	Gram+ and atypicals	Methylation of 23S rRNA, efflux	Used in penicillin allergy; QT prolongation	(17)
Fluoroquinolones	Ciprofloxacin, Levofloxacin	Inhibit DNA gyrase and topoisomerase IV	Broad (Gram– > Gram+)	Target mutations, qnr genes, efflux	Risk of tendon rupture; increasing resistance	(13)
Sulfonamides + TMP	Sulfamethoxazole + Trimethoprim	Block folate synthesis at two steps	Broad (esp. UTIs, PJP)	Target alteration, plasmid-mediated resistance	Synergistic combo; used for PJP, UTIs	(18–20)
Lipopeptides	Daptomycin	Insert into membrane → depolarization and ion leakage	Gram-positive (MRSA, VRE)	Membrane charge alteration	Inactive in pneumonia (surfactant inhibition)	(16)
Nitroimidazoles	Metronidazole	Generate free radicals that damage DNA	Anaerobes, C. difficile	Decreased uptake, nitroreductase mutation	Avoid alcohol (disulfiram-like reaction)	(13)
Oxazolidinones	Linezolid, Tedizolid	Bind 50S, prevent initiation complex formation	Gram-positive (MRSA, VRE)	23S rRNA mutations, L3 protein alterations	Monitor for myelosuppression; reserved for resistant cases	(18–20)
Polymyxins	Colistin, Polymyxin B	Disrupt outer membrane via LPS binding	Gram-negative (MDR)	MCR-1 gene (modifies lipid A), efflux	Last-resort agent; high nephrotoxicity	(13, 16)
Rifamycins	Rifampin, Rifabutin	Inhibit RNA polymerase → block transcription	Gram-positive, TB	rpoB gene mutations	Potent inducer of CYP450; orange discoloration	(17)
Lincosamides	**Clindamycin**	Bind 50S → inhibit peptide bond formation	Gram-positive, anaerobes	erm-mediated methylation	Risk of C. difficile colitis	(18–20)

Overview of major antibiotic classes, their mechanisms of action, clinical spectrum, resistance mechanisms, and key therapeutic considerations. This table provides a rapid reference for both established and emerging resistance determinants across clinically important drug classes.

ESBL, extended-spectrum β-lactamase; MRSA, methicillin-resistant Staphylococcus aureus; VRE, vancomycin-resistant Enterococcus; PBPs, penicillin-binding proteins; PJP, Pneumocystis jirovecii pneumonia; MDR, multidrug-resistant; LPS, lipopolysaccharide; CYP450, cytochrome P450; TB, tuberculosis; UTIs, urinary tract infections; TMP, trimethoprim.

See text for further details.

Protein synthesis inhibitors take advantage of ribosomal divergence by binding to the 30S subunit to cause mRNA misreading, blocking aminoacyl-tRNA entry, or stopping elongation at the 50S peptidyl transferase center. Resistance through erm-mediated methylation and active efflux pumps (Mef, Msr) is now common (17, 21). Fluoroquinolones (DNA gyrase/topoisomerase IV inhibitors) and rifamycins (RNA polymerase inhibitors) are examples of nucleic acid-targeting drugs that work against a wide range of bacteria. However, they lose their effectiveness when the target site mutates (gyrA, parC, rpoB) or when plasmid-borne Qnr proteins are present (13, 22, 23).

Sulfonamides and trimethoprim are metabolic inhibitors that block tetrahydrofolate synthesis enzymes that are not present in humans. When used together (co-trimoxazole), they are still helpful in treating urinary tract and Pneumocystis infections, even though newer drugs mostly replace them (18–20). New classes of oxazolidinones (like linezolid), pleuromutilins, and LptD inhibitors show how structure-guided innovation can work against Gram-negative bacteria that are very resistant to antibiotics in preclinical models (1). Understanding these mechanisms is essential for using antibiotics wisely and for discovering new antibiotics, but the rise of β-lactamases, efflux pumps, target modifications, and toxicities shows how badly we need immunotherapeutic strategies that use the body’s defenses to work with and possibly even beat conventional antibiotics (9, 11, 12).

## 4 Antimicrobial resistance: mechanisms and global therapeutic challenges

**Figure 2:**
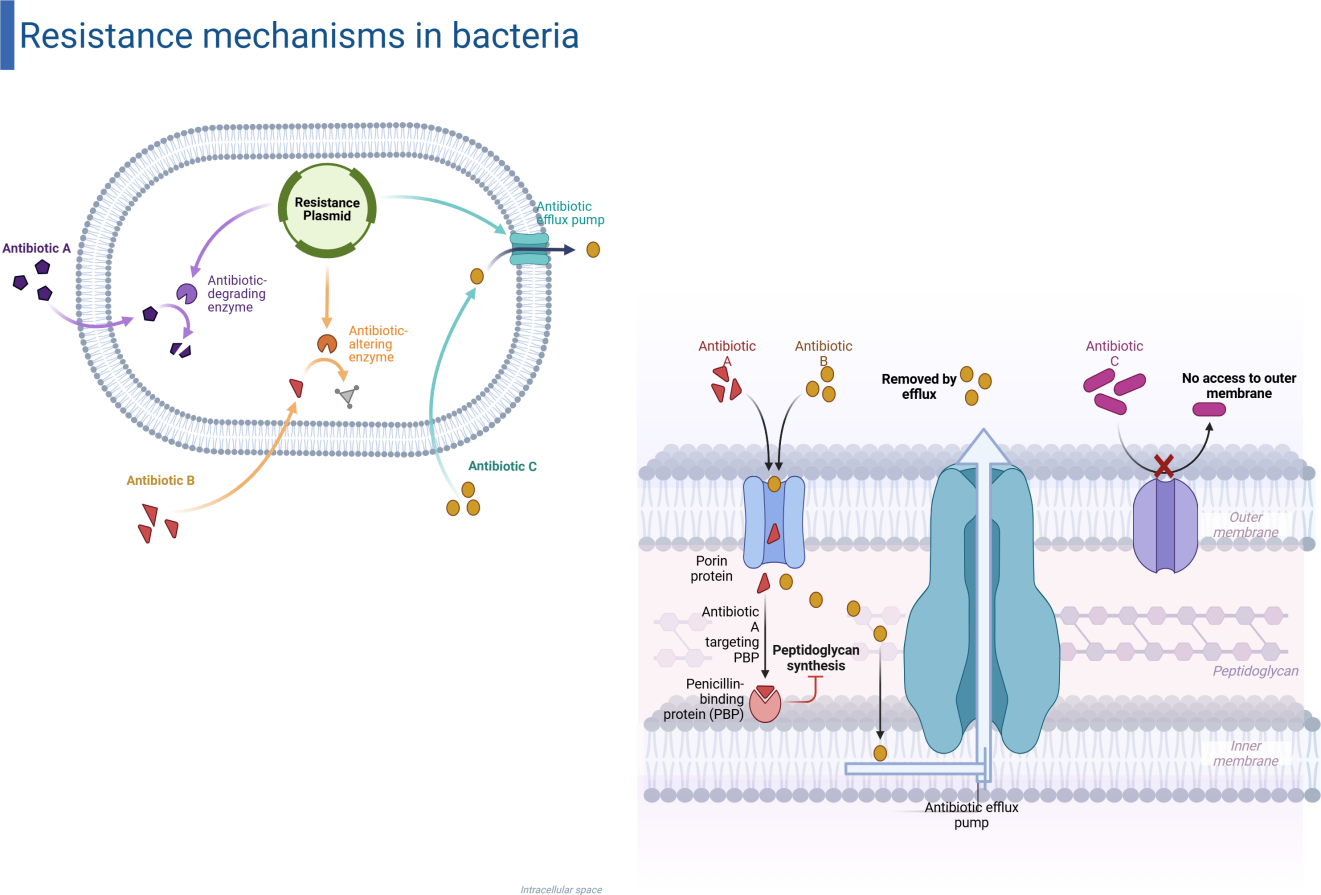
Resistance mechanisms in gram-negative bacteria. Overview of primary innate and acquired antibiotic resistance mechanisms in Gram-negative bacteria. Plasmid-mediated resistance: antibiotic-degrading enzymes (purple), antibiotic-modifying enzymes (orange), and efflux pumps (teal) diminish intracellular drug levels. (Correct) Envelope-level barriers: (1) diminished absorption due to porin loss or mutation, (2) active efflux of internalized drugs, and (3) exclusion by an intact outer membrane, collectively obstructing antibiotics from accessing their targets.

AMR happens when bacteria develop defenses that make antibiotics less effective (8, 24). Intrinsic resistance in Gram-negative outer membranes and constitutive efflux pumps (AcrAB-TolC, MexAB-OprM) makes it harder for drugs to build up inside cells (15, 21). Horizontal gene transfer of plasmids, integrons, transposons, and bacteriophages that carry β-lactamases, aminoglycoside-modifying enzymes, and target-modifying methylases is how acquired resistance spreads (23). Point mutations in gyrA, parC, and rpoB make fluoroquinolone and rifampicin binding even weaker, but compensatory changes often make up for this (22, 25). Biofilm formation embeds persister cells in an extracellular matrix, creating phenotypic tolerance that underpins chronic and device-associated infections (9, 24). **Figure 2** schematizes these multifaceted resistance strategies in Gram-negative pathogens.

The global resistance crisis could undo medical progress as last-resort treatments fail and new antibiotics are slow to come out. There were only three new antibiotics between 2017 and 2022 because of economic disincentives and scientific bottlenecks (10, 26). Bacteriophage therapy can kill specific bacteria without harming other bacteria. Phage cocktails have been shown to reduce Pseudomonas aeruginosa biofilms and work with antibiotics in cystic fibrosis models (27, 28). Host-directed immunotherapies boost both innate and adaptive defenses. For example, monoclonal antibodies like bezlotoxumab for C. difficile neutralize toxins, conjugate vaccines like PCV20 restore serotype coverage, and checkpoint inhibitors like PD-1/PD-L1 blockade boost T-cell responses in tuberculosis models (12, 29). Rapamycin and IFN-γ therapy speed up the removal of pathogens inside cells, while cytokine modulators fine-tune the inflammatory response (11, 30). **Table 3** catalogues these immunotherapeutic modalities.

**Table 3:** FDA-approved bacterial immunotherapies.

Therapy name	Type	Target	Mechanism of action	Indication	Approval year	Key clinical outcomes	Reference
Bezlotoxumab	Monoclonal Antibody	*Clostridioides difficile* Toxin B	Neutralizes toxin B, preventing epithelial damage	Prevention of *C. difficile* recurrence in adults	2016	38% reduction in recurrence rates with antibiotics	(31)
Raxibacumab	Monoclonal Antibody	*Bacillus anthracis* Protective Antigen (PA)	Binds PA toxin, inhibiting lethal toxin activity	Inhalational anthrax (prophylaxis and treatment)	2012	64% survival improvement in animal models	(32)
Obiltoxaximab	Monoclonal Antibody	*Bacillus anthracis* Protective Antigen (PA)	Neutralizes PA toxin to block lethal toxin effects	Treatment/prophylaxis of inhalational anthrax	2016	83–100% survival in animal models	(33–35)
IVIG (Off-label use)	Polyclonal Antibodies	Broad-spectrum bacterial exotoxins	Provides passive immunity by neutralizing circulating toxins	Adjunct for streptococcal toxic shock syndrome	N/A	Reduced mortality in streptococcal TSS	(36)

Summary of FDA-approved bacterial immunotherapies, their types, molecular targets, mechanisms of action, indications, approval years, and key clinical outcomes. This table provides an at-a-glance reference for currently authorized monoclonal and polyclonal antibody therapies targeting major bacterial toxins or pathogens.

IVIG, intravenous immunoglobulin; PA, protective antigen; TSS, toxic shock syndrome; FDA, U.S. Food and Drug Administration; N/A, not applicable.

## 5 Bacterial immunotherapy: mechanisms and innovations

**Figure 3:**
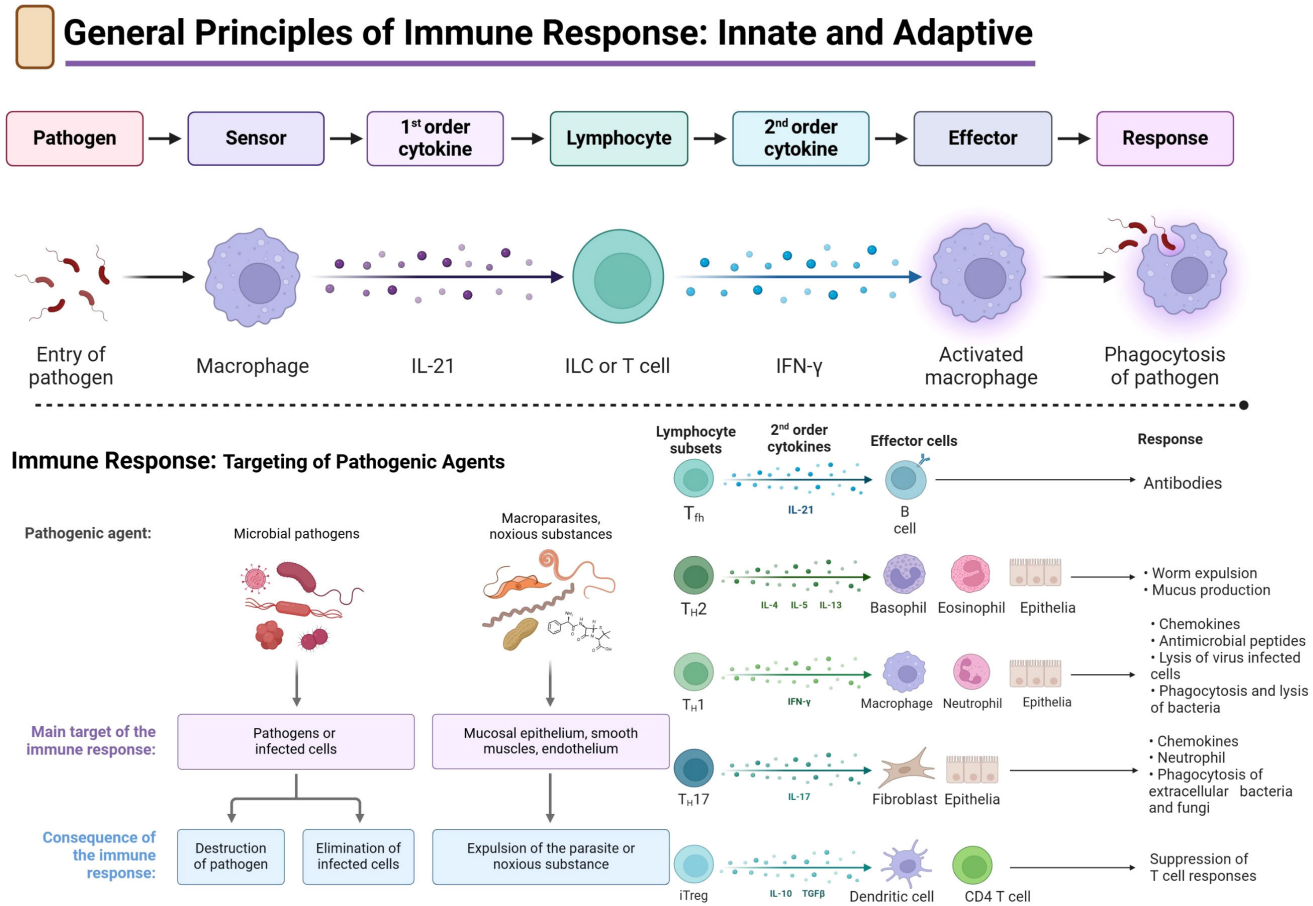
General principles of the innate and adaptive immune response. Schematic representation of the biphasic immunological response to bacterial infection. The upper panel depicts pathogen recognition by innate sensors (e.g., macrophages), subsequent release of primary cytokines (e.g., IL-21), activation of lymphocytes, generation of secondary cytokines (e.g., IFN-γ), recruitment of effector cells, and final eradication of pathogens by phagocytes. The lower panel illustrates the differentiation of CD4^+^ T-cell subsets (T_h_1, T_h_2, T_h_17, T_fh, iTreg), their specific cytokines, major targets of effector cells, and the consequent immunological activities against various infections.

Bacterial immunotherapy utilizes and reconfigures host immunity to eliminate pathogens, serving as a strategic adjunct to antibiotics (11). Modulation of innate immunity leverages pattern recognition receptors, including TLRs and NLRs, to activate phagocytes and cytokine secretion. Synthetic TLR agonists, such as CpG oligodeoxynucleotides for TLR9 and monophosphoryl lipid A for TLR4, improve pathogen clearance in models of pneumonia and sepsis (37, 38). NLRP3 inflammasome inhibitors (MCC950) balance pyroptosis and inflammation (39), while cGAS–STING activation via cyclic dinucleotide analogs drives type I interferon responses essential for intracellular bacteria like Listeria and Chlamydia (40). Monoclonal antibodies bind toxins or surface antigens, bezlotoxumab against C. difficile toxin B and suvratoxumab targeting S. aureus α-toxin, achieving toxin neutralization and opsonophagocytosis in clinical trials (31, 41). Checkpoint inhibitors (anti/PD-1/PD-L1) reverse T-cell exhaustion in tuberculosis models, restoring cytokine production and bacterial control (12, 42). Refer to **Figure 3** for key innate and adaptive pathways and **Table 4** for mechanistic platforms and development stages.

**Table 4:** Key mechanistic platforms in bacterial immunotherapy and their developmental status.

Platform	Mechanism	Key example	Development stage	Reference
Innate Immune Agonists	TLR and inflammasome activation	CpG oligodeoxynucleotides (TLR9); MCC950 (NLRP3)	Preclinical/Phase I	(39)
cGAS–STING Agonists	Type I IFN induction via cytosolic DNA sensing	Synthetic cyclic dinucleotide analogs	Preclinical	(40)
Monoclonal Antibodies	Toxin neutralization and opsonization	Bezlotoxumab (*C. difficile* toxin B)	Approved/Phase III	(31)
Checkpoint Inhibitors	Reversal of T-cell exhaustion	Anti–PD-1 in tuberculosis models	Preclinical/Phase I	(42)
CAR-Engineered Myeloid Cells	Antigen-specific phagocytosis	CAR-macrophages against *P. aeruginosa* biofilms	Preclinical	(43)
CRISPR-Based Antimicrobials	Sequence-specific bacterial killing or gene silencing	CRISPR–Cas9 phagemids targeting resistance genes	Preclinical	(44)

Overview of key mechanistic platforms in bacterial immunotherapy, highlighting representative examples and their current stage of clinical or preclinical development. This table summarizes innovative modalities ranging from innate immune agonists to genetic antimicrobials, emphasizing the translational continuum from bench to bedside.

TLR, toll-like receptor; NLRP3, NOD-like receptor family pyrin domain containing 3; IFN, interferon; PD-1, programmed cell death protein 1; CAR, chimeric antigen receptor; FDA, U.S. Food and Drug Administration; Preclinical, not yet tested in humans.

Beyond soluble factors, engineered cellular therapies and gene-based antimicrobials are transforming the field. CAR-macrophages programmed to recognize P. aeruginosa biofilm antigens improve phagocytosis and inflammatory signaling in murine lung models. (43), and CRISPR–Cas9 phagemids selectively eliminate multidrug-resistant strains in preclinical studies (44). mRNA vaccines validated in viral settings are being tailored to bacterial targets (N. gonorrhoeae, K. pneumoniae), eliciting robust humoral and cellular immunity (45, 46). Nanocarrier systems co-deliver immunomodulators and antibiotics, achieving synergistic biofilm disruption in MRSA and P. aeruginosa models (47). As these innovations move toward clinical use, integrating real-time diagnostics and patient stratification will be essential to overcoming immune heterogeneity and off-target effects, thereby turning mechanistic potential into effective, precision anti-infective therapies. (48).

## 6 Challenges in antibacterial therapeutic development

Inherent bacterial defenses and pharmacological limitations have thwarted decades of antibiotic discovery. Gram-negative pathogens possess a double‐membrane architecture and robust efflux systems (AcrAB-TolC, MexAB-OprM) that actively expel diverse compounds, reducing intracellular drug accumulation (49, 50). Biofilms further shield bacteria within extracellular matrices, fostering profound tolerance to both antibiotics and immune effectors and impeding penetration of even novel agents (24). Meanwhile, many promising compounds fail in preclinical or early clinical stages due to suboptimal pharmacokinetics, poor tissue distribution, and unexpected host toxicities. These challenges are worsened by the narrow therapeutic windows needed to protect commensal microbiota and prevent immunosuppression (16).

Beyond scientific challenges, systemic barriers significantly hinder antibacterial innovation. Antibiotics’ short-course regimens, stewardship constraints, and generic competition result in low returns on investment, discouraging extensive pharmaceutical engagement (26). Regulatory requirements that favor broad-spectrum superiority trials over pathogen-specific or adjunctive therapies make it even harder to get things approved. These things hinder antibiotic research and development, highlighting the need for alternative approaches. Bacterial immunotherapy, which includes monoclonal antibodies, engineered immune cells, and targeted delivery platforms, could change everything. However, it still has to deal with the same biological, pharmacological, and regulatory issues to have an effect in the real world.

## 7 Integrated strategies: aptamers, nanotechnology, and beyond

Innovative precision platforms, including aptamer-guided targeting, nanotechnology, CRISPR-based antimicrobials, and engineered phages, are revolutionizing the treatment of bacterial infections by circumventing the limitations of conventional drugs, such as inadequate specificity, toxicity, and the inability to eradicate biofilms or intracellular pathogens (44, 51). Aptamers are concise, single-stranded oligonucleotides that exhibit strong binding affinity to other molecules. Initially employed for diagnostics, they are currently utilized for treatments due to their ease of production and lack of immunological reaction. Attaching to lipid or polymeric nanoparticles enhances medication delivery into Pseudomonas aeruginosa biofilms, resulting in more bacterial eradication with reduced side effects (51). Aptamer-encapsulated, membrane-coated nanoparticles have effectively neutralized potent neurotoxins such as saxitoxin *in vivo*, showcasing the adaptability of this method (52).

**Figure 4:**
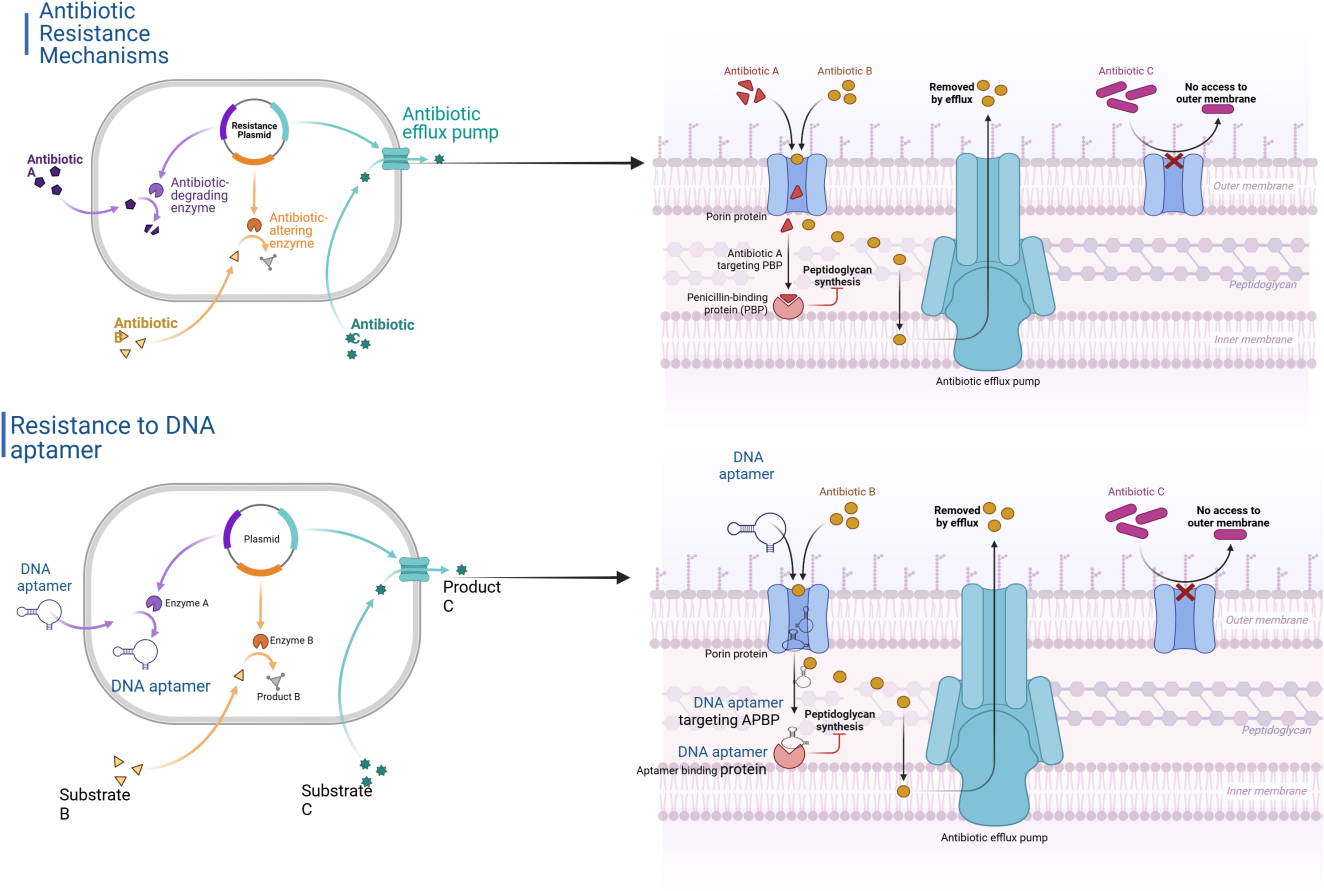
Conventional antibiotic vs. DNA-aptamer resistance mechanisms. The upper panels illustrate Conventional Resistance, where resistance plasmids encode degrading (purple) and modifying (orange) enzymes, together with efflux pumps (teal). These components jointly neutralize antibiotics A–C by enzymatic inactivation, target mutation, diminished absorption, and active efflux. The lower panels depict DNA-Aptamer Resistance. DNA-aptamers (blue) associate with critical resistance enzymes or penicillin-binding proteins (APBP), redirecting metabolite flow from substrates B to products C. Nonetheless, these systems equally face obstacles with efflux and absorption.

Nanocarriers, especially lipid nanoparticles (LNPs) that have been shown to work in mRNA vaccines, now carry CRISPR–Cas9 systems and immunomodulators directly to infection sites. They turn off resistance genes (like mecA in MRSA) and make animals more sensitive to antibiotics (53). CRISPR-Cas antimicrobials and CRISPR interference (CRISPRi) can kill specific sequences or turn off genes temporarily. They can get rid of multidrug-resistant strains while leaving commensals alone (44). Engineered bacteriophages that have enzymes that break down biofilms or ligands that boost the immune system make it even easier for S. aureus and P. aeruginosa biofilms to get through and be removed (54). Lastly, theranostic platforms that use nanoparticles with aptamers or antibodies to find pathogens and destroy them with heat and light show how diagnosis and therapy can be combined into one construct (55). These combined strategies mark the beginning of a new era of particular, flexible, and synergistic anti-infective treatments that are ready to break through long-standing resistance mechanisms (**Figure 4**).

## 8 Integrated precision strategies: aptamers, CRISPR, and nanoplatforms in immunotherapy

IPS deploy two or more PBI modalities together to overcome biofilms, intracellular reservoirs, and multidrug-resistant (MDR) traits. A new generation of aptamer-nanoparticles, programmable gene-editing systems, and multifunctional carriers illustrates this concerted approach.

Aptamer-guided nanocarriers. Structured oligonucleotide aptamers can be anchored to carbon nanomaterials. These constructs penetrate mixed Pseudomonas aeruginosa–Staphylococcus aureus biofilms and enhance antibiotic killing (56). Enzyme-functionalised silver nanoparticles disrupt dual-species matrices and reduce biomass (57). C-phycocyanin-coated silver particles further suppress biofilm mass and virulence-gene expression in resistant strains (58). Aptamer-gold nanorods add theranostic capability: they image pathogens optically and ablate them by photothermal heating (55, 59).

CRISPR cargos and engineered phages. Lipid or polymer nanoparticles can ferry CRISPR–Cas9 that deletes mecA in methicillin-resistant S. aureus, restoring β-lactam susceptibility both *in vitro* and *in vivo* (53, 60). Conjugating guide RNAs to pathogen-specific aptamers restricts editing to infection sites and limits off-target effects (61). Engineered bacteriophages supply matrix-degrading enzymes or immune-activating ligands, loosening entrenched S. aureus and P. aeruginosa biofilms (54).

Microbiome-modulating nanocarriers. Muco-adhesive particles that deliver probiotics such as Lactobacillus reuteri, or prebiotic substrates, reduce Clostridioides difficile colonisation and toxin levels in murine gut models (45). Together, these IPS platforms fuse targeted diagnostics, gene editing, immune modulation, and conventional antibiotics into modular, programmable systems that out-flank entrenched antimicrobial resistance.

## 9 Clinical applications and translational development

Different host responses, a wide range of pathogens, and complicated trial design make it hard to put bacterial immunotherapies into practice (62, 63). Still, several candidates show that they can work in the real world. Monoclonal antibodies have made the most significant impact. For example, bezlotoxumab, which targets C. difficile toxin B, stops the disease from coming back in patients who are at high risk (31). Suvratoxumab (MEDI4893) cuts the rate of ventilator-associated pneumonia by 32% in Phase 2 (41), AR-301 boosts the cure rate for hospital-acquired pneumonia by 40% when combined with antibiotics (41). Checkpoint inhibitors (anti–PD-1) look like they could work against multidrug-resistant tuberculosis; in Phase 1, they cut the amount of sputum by 50%. However, there are risks of immunopathology and reactivation of latent infections (42, 64). In early trials (ACTG A5361), cytokine superagonists (N-803) helped NK cells get rid of latent M. tuberculosis, but safety and dosing are still being looked into.

Vaccine platforms are getting better very quickly. mRNA candidates against K. pneumoniae, A. baumannii, and N. gonorrhoeae use a modular design to make it easy to change things rapidly, but getting strong mucosal immunity is still a problem (45, 65). In Phase 2b, Pfizer’s SA4Ag four-antigen S. aureus vaccine was 80% effective (66). The VLA84 toxoid vaccine cut the number of C. difficile infections in older people by 72% (67). Cell-based therapies like CAR-macrophages and neutrophils are very effective at getting rid of P. aeruginosa biofilms in mice (43) However, they are challenging to prepare quickly and have numerous rules to follow for acute infections.

Combination strategies that use immunotherapies with antibiotics or nanocarriers together show that they work better together to lower the levels of P. aeruginosa and MRSA (47). At the same time, real-time diagnostics help doctors decide how to group patients based on their pathogen load and immune status (48). In the future, success will depend on multi-modal regimens that are tailored to the biology of the pathogen and the host’s immunity, strong immune profiling, and adaptive trial designs with multiple endpoints (such as eradication, immune restoration, and relapse prevention) to show clear clinical benefit and get regulatory approval.

## 10 FDA-approved bacterial immunotherapies

Monoclonal antibodies are the most successful immunotherapy for bacterial infections that have been approved so far. In 2016, bezlotoxumab, a human monoclonal antibody that targets Clostridioides difficile toxin B, was approved. It is still the only licensed product. When used with standard antibiotics, it has been shown to lower the number of times high-risk patients get C. difficile infection again by 38% (31). Immunostimulatory therapies like granulocyte-macrophage colony-stimulating factor (GM-CSF or sargramostim) are also used to boost the body’s defenses, in addition to antibody-drug conjugates. GM-CSF was first approved to treat neutropenia caused by chemotherapy, but it has also been shown to help in cases of infection. In diabetic foot infections (DFIs), adding GM-CSF therapy made a big difference in how quickly the bacteria cleared up and the wounds healed. Clinical data showed that 65% of wounds closed up compared to 40% with just antibiotics (47). More research backs up its ability to change the immune system in infections that affect the lungs and the whole body (68, 69). Passive immunization with intravenous immunoglobulin (IVIG) has also been shown to be off-label in infections caused by nasty toxins. In cases of streptococcal toxic shock syndrome, IVIG therapy cut the death rate in half, showing how it can help control hyperinflammatory responses during invasive bacterial disease (36).

## 11 Discussion

### 11.1 Integrating precision bacterial immunotherapy with antimicrobial stewardship

PBI encompasses host-directed interventions—such as toxin-neutralising monoclonal antibodies (mAbs), CRISPR antimicrobials, and engineered phagocytes—that eradicate infection while sparing the commensal microbiota. Integrated IPS combine complementary PBI modalities (e.g., mAbs delivered alongside CRISPR phagemids or aptamer-guided nanocarriers) to neutralise multiple bacterial defences in parallel. Late-phase clinical data illustrate the single-agent promise of PBI: bezlotoxumab lowered recurrent Clostridioides difficile infection by 38% in high-risk adults (31), and suvratoxumab reduced Staphylococcus aureus ventilator-associated pneumonia by 32% (41). Yet the accelerating pace of AMR and the stagnating antibiotic pipeline demand adaptable IPS tailored to pathogen–host contexts (26).

### 11.2 Assessing reliability across translational stages

Our mechanistic taxonomy spans proof-of-concept studies through phase III trials, revealing striking variability in evidentiary strength. Elegant pre-clinical demonstrations—such as sequence-specific CRISPR phagemids that eliminated multidrug-resistant S. aureus *in vitro* (44)—often rely on a single strain or modest sample size, limiting generalisability. Early-phase cellular approaches, including chimeric antigen receptor (CAR) macrophages that clear Pseudomonas biofilms in murine lungs (43), still face questions about off-target inflammation and manufacturing scalability. By contrast, late-stage toxin-neutralising antibodies provide robust safety datasets but may be undermined by antigenic variation and biofilm shielding.

### 11.3 Mechanistic synergies and delivery bottlenecks

IPS frameworks underscore the complementarity of distinct modalities. CRISPR nucleases can excise resistance cassettes to restore antibiotic sensitivity (53), whereas aptamer-functionalised lipid nanoparticles intensify antibiotic exposure within dense biofilms (3) Photothermal aptamer–gold nanorods add an orthogonal, light-triggered killing mechanism (55). Poor penetration into necrotic tissue and host phagolysosomes remains a bottleneck; host-targeted small molecules such as the NLRP3 inhibitor MCC950 illustrate how dampening excessive inflammation can improve antibiotic access without selective pressure (39).

### 11.4 Research gaps and regulatory considerations

Key knowledge gaps include standardised potency assays for engineered phage cocktails (27), long-term microbiome effects of CRISPR cargo delivery, and validated biomarkers for adaptive IPS dosing. Regulatory pathways are unsettled because many PBI products straddle drug, biologic, and device classifications, complicating approval and reimbursement. Economic disincentives—already acute for small-molecule antibiotics—may be even steeper for complex biologics that target niche indications.

### 11.5 Outlook and future directions

Precision bacterial immunotherapy is poised to shift infectious-disease care from microbe-centred eradication to host-focused modulation, holding promise for durable AMR control while sparing commensals. Early clinical victories validate the principle but expose the limitations of single-antigen agents, which can be evaded by antigenic drift or embedded within biofilms (31, 41). The field is advancing toward IPS that pair CRISPR phagemids excising resistance genes (44), CAR-engineered phagocytes penetrating biofilms (43), and aptamer-guided nanocarriers concentrating payloads at infection foci (51)—all guided by real-time, multi-omic diagnostics for patient stratification (26). “Safety-by-design” measures such as self-limiting genetic circuits and inflammasome inhibitors (39) will be crucial, as will harmonised manufacturing standards and reimbursement frameworks that build on phage-therapy precedents (27). If scientific, regulatory, and economic levers align, IPS could evolve from salvage options to frontline standards within the next decade (**Figure 1**).

## 12 Conclusion

Precision bacterial immunotherapy reframes infectious-disease treatment by redirecting the therapeutic focus from pathogen eradication to host-centred modulation. By weaving together monoclonal antibodies, CRISPR-based antimicrobials, engineered immune cells, and aptamer-enabled nanocarriers, our mechanistic taxonomy demonstrates how rationally orchestrated combinations can dismantle biofilms, silence resistance genes, restore antibiotic susceptibility, and temper damaging inflammation. Mapping each modality along a translational continuum highlight both the clinical maturity of toxin-neutralizing antibodies and the emerging promise of gene-editing and nano-delivery strategies, while underscoring persistent challenges in tissue penetration, immune profiling, and regulatory harmonisation. Realising the full potential of PBI will demand coordinated investment, adaptive clinical trial designs, forward-thinking stewardship policies, and regulatory innovation, but the payoff is substantial: durable, host-tailored interventions capable of outpacing bacterial evolution and safeguarding anti-infective efficacy for the next generation.
